# The Role of SES in Preadolescence: Understandings and Group Evaluations based on Income, Education, and Occupation

**DOI:** 10.1007/s10964-024-02018-2

**Published:** 2024-05-29

**Authors:** Iris Boer, Fenella Fleischmann, Jochem Thijs

**Affiliations:** 1 ERCOMER, Department of Interdisciplinary Social Sciences, Utrecht University, Padualaan 14, 3584 CH Utrecht, The Netherlands; 2 Department of Sociology, University of Amsterdam, Nieuwe Achtergracht 166, 1018 WV Amsterdam, The Netherlands

**Keywords:** Socioeconomic status, Group evaluations, Socioeconomic understanding, Preadolescence

## Abstract

Socioeconomic status (SES) predicts many facets of preadolescents’ life opportunities, yet little is known about how children perceive SES and whether it plays a role in their group evaluations. This study examined preadolescents’ socioeconomic understandings and biases (investigated presenting fictitious peers varying in SES), while separating the three SES-indicators income, education and occupation. Five classes (Grade 4–6) with 89 students (*M*age = 10.44, *SD* = 0.93; 40% female) participated. Overall, preadolescents understood that the SES indicators income, education and occupation are related to each other. At the same time, they differentiated between the indicators in their group evaluations; they showed a positive bias for peers with high-educated parents, whereas for occupation and income there was no clear overall bias. This shows that differences between SES indicators are meaningful for children, which emphasizes the importance to distinguish between specific SES-indicators when studying the role of SES for preadolescents’ social life.

## Introduction

There is ample evidence that socioeconomic status (SES) predicts many facets of children’s life opportunities, such as their health outcomes (Duncan et al., 2015), academic attainment (Lawson et al., 2018), and life satisfaction (Elgar et al., 2017). SES consists of multiple dimensions, or indicators, which have unique effects on children’s lifes (i.e., income, education, and occupation; Duncan & Magnuson, 2003), but relatively little is known of children’s own understandings and awareness of SES and its multidimensionality. Furthermore, although children’s family SES is related to getting bullied (e.g., Contreras et al., 2015), and even though children regard SES-based social exclusion as morally wrong (e.g., Gonül et al., 2023), little is known about their evaluations of different SES groups. While there is evidence that children show socioeconomic bias due to status differences or stereotypes (Mistry et al., 2015), existing research has merely focused on the dimension of income and not considered education and occupation. Hence, the current study had two broad aims. The first one was to investigate children’s understandings of the concept of SES, by examining the accuracy with which they estimated the SES of their own family, and their perceptions of the relations between the SES indicators education, income, and occupation. The second goal was to research how children evaluated (hypothetical) peers with high- and low- socioeconomic family backgrounds, and whether their biases were similar across the different indicators income, occupation, and education.

The present study was conducted among a sample of Dutch preadolescent children (9–12 years of age). During preadolescence, more sophisticated understandings of inequality are acquired (for a review, see Dickinson et al., 2023) and children of this age have the cognitive skills to develop nuanced and complex group attitudes (Aboud & Amato, 2001). Unlike the U.S. and the U.K., where most work on this topic has been done, there is comparatively less economic inequality in the Netherlands, and a weaker endorsement of meritocracy (i.e., the belief that status in society is based on ability and effort; Mijs, 2018). At the same time, high-educated people tend to have a higher social standing in this country (Kuppens et al., 2018), and there is currently a lot of attention for this with debates about how to refer to low- and high-educated people. Children could be aware of these status differences, which might influence how they see the world around them and evaluate different groups. Compared to other countries, children’s group evaluations based on parents’ educational level may therefore be more biased. Further, the participating children were students in grades 4–6, which in the Dutch school system implies a period of important educational decision making that has repercussions for their later own SES^1^ - making differences in educational level increasingly salient and relevant to their own lives.

### Theoretical Perspectives

SES is one of the most widely researched constructs in the social sciences (Mistry et al., 2015). It indexes people’s hierarchical position via relatively objective indicators of prestige, power, and control over resources (Diemer & Ali, 2009). Although there is debate about how to conceptualize and measure SES (Diemer et al., 2013), there is agreement that it involves income, educational level, and occupational status (Duncan & Magnuson, 2003). These three indicators do correlate with each other, but they are also unique, and each one measures a substantially different aspect of SES (Erola et al., 2016). Although these indicators are sometimes regarded as interchangeable proxies for SES, contemporary studies increasingly consider all of them separately (see for example Klein et al., 2020). This research shows that the different SES indicators appear to have supplementary, unique effects on children’s outcomes (e.g., Bukodi & Goldthorpe, 2013). The current study follows this approach and takes the SES indicators separately into account to study children’s understandings and group evaluations. Because it concerns preadolescent children, the focus was on their parents’ income, years of education, and occupation – referred to as family SES.

There are two broad and complementary theoretical perspectives that together could explain how children come to develop an understanding of SES (see Enesco & Navarro, 2003). Following a *Cognitive Developmental Perspective* (CDP), children could discover what SES means by reasoning and thinking based on their own experiences and cognitive abilities. For example, at a certain age, children might come to the “logical” conclusion that, because they have better qualifications, people with higher educational levels have more access to higher status jobs, and this conclusion might be facilitated by observing the particular situation of their parents. At the same time, following Moscovici’s *Social Representations Theory* (SRT; Moscovici & Hewstone, 1983), children might also come to learn about SES by being exposed to common knowledge about it in their social context. Thus, they might simply know that cleaners earn less than bankers because “everybody knows this”.

The first theoretical perspective (CDP) is based on the work by Piaget and focuses on how children construct knowledge about social reality based on their experiences and according to their stage of development (see for example, Enesco & Navarro, 2003). It holds that children form their own images, ideas, and theories as a result of their explorations and developing thinking capacity (Furth, 1980). Social knowledge is thus partly individual knowledge, discovered through own reasoning and personal experiences. The second theoretical framework (SRT) considers social representations as mechanisms through which children learn about the world around them. Social representations are common understandings about aspects of society – created and recreated through interaction and communication between people (Moscovici & Hewstone, 1983). Because children are born into a social context already filled with social representations, they are assumed to gradually reconstruct those representations in their minds, and this can explain why children from similar environments have similar understandings of social reality (see Verkuyten & Masson, 1994). Social knowledge can therefore also take the form of shared, common-sense understandings obtained by being part of the same social context.

Importantly, both theoretical perspectives can also explain how children evaluate different SES groups. For example, Bigler and Liben’s Developmental Intergroup Theory (2007), in line with the CDP, specifies how cognitive abilities such as classification skills play a role in prejudice development, and this theory has been successfully applied to children’s SES relations (e.g., by studying how children attribute traits to people with a different family SES; Mistry et al., 2021). Likewise, children’s stereotypes about socioeconomic groups may be considered as specific social representations, being part of the societal context they grow up in (see Vandebroeck, 2021).

### Preadolescents’ Understandings of Family SES

#### Estimations of own family SES

A good starting point for examining children’s understandings of SES is to test their ability to estimate the SES of their family (cf., Mistry et al., 2015). Empirical research to date has focused on subjective social status (SSS, see Diemer et al., 2013) which involves the positioning of oneself or one’s family on a social ladder ranging from the least favorable versus the most favorable position. Goodman et al. (2015) used a ladder that referred to all three indicators of SES, and found that the SSS scores of adolescents and their mothers were only moderately related (*r* = 0.38). Research among children has focused on the dimension family wealth in particular, and yielded some but inconsistent evidence that they can accurately estimate their SES. For example, one study found that children’s SSS ratings were correlated with their parents’ actual income (*r* = 0.64) and their parents’ SSS (*r* = 0.56) (Mistry et al., 2015), and another study found that 75% of a sample of 10-year-olds could accurately indicate whether their parents’ income was above or below the median of their zip code (Peretz-Lange et al., 2022). However, Ackerman and Elenbaas (2023) found no significant correlation between children’s SSS ratings and parent-reported income.

Overall, the available research suggests that children are to some extent aware of their parents’ income, and to a lesser extent of their parents’ education (Engzell & Jonsson, 2015). Based on the CDP, one might expect that children estimate their own parents’ education less accurately compared to their parents’ income and occupation. Children probably have more concrete experiences with the latter at home. For example, parents might talk more often about their work and whether they have money to spend on something, compared to their education, which they typically followed in the past. Also, parental education is a much more abstract indicator, and traditional theories of cognitive development argue that abstract thinking still develops in late childhood (Simons & Keil, 1995). Accordingly, it was expected that children were more accurate in estimating their parents’ income and occupation (i.e., more concrete indicators of SES following the CDP) than their parents’ education (Hypothesis 1).

#### Relations between SES indicators

Both the CDP and SRT lead to the prediction that children see a stronger relation between occupation and income, compared to the relations between occupation and education and income and education. Following SRT, people’s occupation and income is presumably more prominent in children’s social worlds compared to people’s education. For example, children may discuss their parents’ occupation with others at school, and their family’s income (or at least purchasing power) may become visible during class activities, conversations and assignments. Moreover, occupation and income, and the link between the two, might be more prominently depicted in the media children are exposed to (e.g., tv-series or movies). Because of this, children might have a common-sense understanding of the fact that people with a higher occupational status have higher income. As mentioned, children might have more personal experiences with their own parents’ income and occupation compared to education. For example, parents might talk about receiving money from their work, and mention that because they work there is money to spend. Also, children of parents without independent means (e.g., generational wealth) may experience the consequences of unemployment or underemployment on their parents’ purchasing power. Because of this, and consistent with the CDP, the relation between income and occupation might be easier to understand and more ‘logical’ for children than the more indirect relations between income and occupation with education.

There is some empirical evidence which suggests that the relation between income and occupation is indeed clear for preadolescent children. One study found that U.S. fifth-graders mainly explained wealth and poverty, respectively, by having a (good) job, and a poor job or no job at all (Sigelman, 2012). The children also referred to (not) working hard and (not) receiving an inheritance in their explanations, but to a lesser extent. Similarly, Mexican and Spanish children aged 10 and 12 indicated work to be the most important reason why people get rich (Enesco & Navarro, 2003). With age, children better understood that it depends on the kind of work you do how rich you will be – and not only on the amount of hours you work. Finally, Belgian children aged 10–12 who got to see pictures of parents with different clothing styles, were able to quite accurately indicate which of the parents had the highest and lowest income, and to match all parents with the right occupations from a provided list (Vandebroeck, 2021).

In contrast, it appears that preadolescent children’s knowledge of educational level in relation to income and occupation is still limited, although developing (see Dickinson et al., 2023). Sigelman (2012) showed that children do not use education in their explanations for wealth. For poverty, children did refer to having low education, but to a lesser extent than work-related factors. Also, Enesco and Navarro (2003) found that children referred considerably less to education compared to work as a reason why people get rich, but with age they did increasingly refer to education as an important factor in getting certain jobs. Overall, based on the CDP and SRT, and empirical work, it was expected that children perceive relations between all SES indicators, but more strongly so between income and occupation (Hypothesis 2).

Furthermore, from both theoretical perspectives it could be argued that children’s knowledge about SES is dependent on their own socioeconomic background. In line with the CDP, children’s own SES could determine the personal experiences they have at home (e.g., children of parents without independent means might talk more at home about money and income in relation to work). Following the SRT, children’s own SES could partly determine their social context with the associated social representations in it (e.g., children in a high-SES context are probably exposed to other kinds of media and activities (i.e., news, books, museums) compared to children in a low-SES context; Klokker & Jæger, 2022). Moreover, following the CDP one could expect that children’s knowledge about SES is dependent on their age (development) as well (Furth, 1980). These possibilities were merely explored in the present study, as there were no clear hypotheses for the moderating effects of children’s own SES, and because the age range was rather limited.

#### Socioeconomic group evaluations

Consistent with both theoretical perspectives (CDP and SRT), preadolescent children can be expected to have specific attitudes toward different SES groups (assessed by asking children about their willingness to befriend fictitious peers with different SES backgrounds; cf., Thijs & Verkuyten, 2023). Thus far, research into children’s SES attitudes has merely focused on income. This research shows that most 9-to-12-year-olds evaluate middle-class children positively (e.g., Mistry et al., 2015) – presumably because they perceive the middle class as the culturally defined ‘normal’ – and prefer to belong to this group (Weinger, 2000). With respect to evaluations of poor and rich peers, the evidence is mixed. On the one hand, there is research showing that preadolescent children – regardless of their own socioeconomic background have mainly negative evaluations of poor children, and positive evaluations of rich children (Gonül, 2020). This was also found when family SES was manipulated through clothes and haircuts (Vandebroeck, 2021). A possible explanation is that children see material wealth as an indicator of social status and generally evaluate people with a low social status more negatively (Nesdale & Flesser, 2001). On the other hand, there is research showing that 8-to-12-year-olds like lower- and middle-SES peers more compared to higher-SES peers (whom they rated rather neutrally) – regardless of their own SES (Ackerman & Elenbaas, 2023). In one study, preadolescent children even showed a relative dislike for rich peers, because they regarded them as greedy and snobby (Elenbaas et al., 2022). In the same vein, adolescents have been found to associate negative attributes more with a high-SES peer than a low-SES peer (with SES manipulated via clothes; Grütter et al., 2021). Given this mixed evidence so far, no clear expectation was formulated in the present study for children’s group evaluations based on parental income. There was also no clear expectation for group evaluations based on occupational status, as this SES indicator has received very little attention in the developmental intergroup literature. However, children were expected to show a positive bias toward peers with high-educated parents (Hypothesis 3). High-educated people tend to have a higher social standing in the Netherlands (Kuppens et al., 2018) and children are probably aware of this.

Possibly, children’s group evaluations are (partly) based on stereotypes they have of high- and/or low-SES families. More specifically, children might refer to characteristics and behaviors of different parents and/or peers (following earlier studies into children’s stereotypes e.g., Elenbaas et al., 2022), when they explain their group attitudes. Also, as differences in SES are often connotated with differences in hobbies (e.g., people from upper-class play the piano), appearances (e.g., poorer people have older clothes; Vandebroeck, 2021), and parenting style (see e.g., Roubinov & Boyce, 2017), children might also refer to this.

An interesting question is whether children’s socioeconomic group evaluations depend on their estimations of their own SES. Existing work on this (see Mistry et al., 2021) has relied on an intergroup perspective – using Developmental Intergroup Theory (DIT; Bigler & Liben, 2007) and Social Identity Development Theory (SIDT; Nesdale, 2004). This intergroup perspective states that children are inclined to categorize themselves and others as group members, and motivated to prefer their ingroup over their outgroup(s) in order to obtain a positive social identity. There is ample support for these notions when it comes to ethnicity, race or gender (see e.g., Verkuyten, 2021). However, unlike those other characteristics, SES indicators are continuous rather than nominal, and this can make it hard to use them as sources of self-categorization. Accordingly, research has found that relatively many children tend to rate themselves as in the middle on scales for subjective social status (SSS; Ackerman & Elenbaas, 2023). This has been referred to as a middle-class bias, and appears to exist irrespective of children’s ‘actual’ position – a finding that has also been obtained by Mistry et al. (2015). Children seem to describe poor and rich people in such extreme terms (e.g., homeless people vs. the president) that they can never identify with them and automatically always place themselves in between. Moreover, children might see ‘the middle’ as being normal or ordinary – which is a desired position to identify with (Mistry et al., 2015). A study that further scrutinized this found that more than four fifths of the participating children positioned themselves near the middle of the SSS scale although they actually had highly different socioeconomic backgrounds (Kostet et al., 2022). Notably, they did not describe this social category of being ‘in the middle’ in similar ways. For example, presumably to present themselves as ‘normal’, some low-SES children constructed a more extreme image of poor people, whereas high-SES children tended to do this for rich people. Thus, children seem to not really express a socioeconomic self-identification when exposed to this scale, but rather a desire to belong to ‘the middle’.

In addition to this, there is not always a clear relation between children’s own SSS and their attitudes toward different SES groups (Ackerman & Elenbaas, 2023). One study even found that lower SSS children had more negative beliefs about poor children than their middle SSS peers (Mistry et al., 2015), indicating a lack of ingroup preference. The last finding does not undermine the intergroup perspective, because distancing the self from a low-status ingroup can also be a strategy to obtain a positive social identity (Brown, 2000). Still, it suggests that the link between children’s own estimations of their family SES and their socioeconomic group evaluations can take various forms, and therefore no specific hypothesis was formulated about it.

## Current Study

SES predicts many facets of (pre-)adolescents life opportunities, yet little is known about preadolescents’ understandings of SES, and their socioeconomic group evaluations with respect to the three indicators income, education and occupation – hence the goal of the present study was to broaden our knowledge about this. Specifically, the current study’s *first aim* was to examine whether children could estimate their own family’s SES. For this aim, it was tested whether children’s estimations were correlated with information provided by their parents – and based on the CDP and available research on children’s SSS, positive correlations for all indicators but more strongly so for income and occupation were expected (Hypothesis 1). The *second aim* was to examine whether preadolescents understood the relations between the SES indicators education, occupation and income. Based on the theoretical underpinnings of CDP and SRT, and empirical work, it was expected that children would perceive relations between all indicators but more strongly so between income and occupation (Hypothesis 2). Related to the second aim, it was explored whether children’s age and own family SES were related to these understandings. Finally, the *third aim* was to examine whether children showed biases in their SES group evaluations. Based on the fact that high-educated people tend to have a higher social standing in the Netherlands, it was expected that children showed a positive bias toward peers with high-educated parents (Hypothesis 3) – but there were no clear expectations for income- and occupational status bias. Related to the third aim, it was explored why children showed biases, and whether children’s own estimations of their family SES indicators were related to this – to test this from an intergroup perspective.

## Methods

### Participants

Participants were 89 students (Grade 4–6) from five primary school classes in a suburban area in the east of the Netherlands. These classes were recruited via the first author’s personal network while substantial variation in students’ family SES between children within classrooms was ensured – which is relatively common for suburban areas (Boterman, 2021). The sample (*M*age = 10.44, *SD* = 0.93; range = 9–12) consisted of 53 boys and 36 girls. 83 students indicated that they were Dutch and 6 students indicated to belong to Dutch as well as another ethnic group. Further, for 80 of these children (89.9%) parents provided information on at least one family SES indicator (see below for specific sample sizes). The study was approved by the Ethics Committee of the Faculty of Social and Behavioral Sciences of Utrecht University (project no. 22-0665). Consent was obtained from parents and students. 29 students (23.6%) who attended the sampled classes were excluded from the study as their parents did not give consent.

### Procedure

Classes were visited in March and April 2023, after (most) parents or caregivers filled out a short online questionnaire about their own SES. During the class visits, students completed a questionnaire using Qualtrics. This took approximately 45 minutes. Children filled out questions related to their SES biases and the relations between the three indicators of SES. For this part of the questionnaire, illustrations of parents were used in which the indicators of SES were manipulated (see Appendix A). To ensure that solely effects of SES were measured, the clothes and/or attributes of the parents were changed to represent the SES indicator, and all other stimuli were kept constant for all parents (e.g., their facial expressions, skin and hair color, gender, and body pose). It was explained to children that the pictures were merely used as an illustration, and that in reality parents could look different from the (white, heterosexual) ones that were depicted. In the last part of the questionnaire, students estimated their own family SES.

### Measurements

#### Relationships between SES indicators

To measure whether children understood the relations between the three indicators of SES (i.e., education – income, education – occupational status, and income – occupational status), 12 items were used. They were displayed in random order and had a 5-point Likert response scale. Each item involved a statement for parents who scored either high or low on an indicator and was shown with a corresponding illustration (see Appendix A). An example item set is, ‘Here you see parents who studied for a long time after high school in their past, what occupation do you think they have?’, with the answer options being ‘An occupation like … (1) gardener, cleaner, or window cleaner, (2) bus driver, bicycle mechanic, or hairdresser, (3) police officer, store owner, or dental assistant, (4) writer, pilot, or teacher, and (5) doctor, dentist, or judge’. A second question asked children to estimate the occupations from the same list after indicating that parents had a low level of education (“did not study after high school”). Moreover, children were asked to indicate the occupations for parents with low and high incomes (described as parents who “can almost never buy expensive things” vs parents who “can buy many expensive things”). Similarly, children were asked four times to rate the income of parents varying in their level of education (low-high) and occupational status (low-high, using answer categories (1) and (5) from the above list of occupations). Finally, they rated the expected level of education of parents with different incomes (low-high) and occupational status (low-high), resulting in a total of 12 items.

The occupational categories used in the items were selected to fit the frame of reference of preadolescent children, and based on the International Standard Classification of Occupations (ISCO) – ISEI-08 (Ganzeboom, 2010). This classification rates the status of all occupations on a scale from 0–100, and the answer options in the occupation items reflected twenty points. In other words, the occupations at the first answer option have a score between 0–20, on the second between 21–40, and so on. At the end of the questionnaire, children were asked to assign different amounts of money bags and books (1–5) to all groups of occupations to check whether they saw them as hierarchically ordered in relation to the other SES dimensions – and children indeed saw this hierarchy supporting the validity of the occupations scale^2^.

#### Estimations of own family SES

Parents were asked to provide their own and—if applicable—their partner’s occupation and highest obtained level of education (ranging from (1) Primary education, to (9) Doctorate). Further, they were asked to give a rough indication of their financial status on a 5-point Likert scale ranging from (1) ‘We struggle to make ends meet financially and this does not always work out’, to (5) ‘We can easily make ends meet and we can save a lot of money’. Parents’ occupations were coded, first by using the ISCO-08 Dutch occupations index (CBS, n.d.). Then, this was recoded using the ISEI-08 (Ganzeboom, 2010) to align with the students’ answer categories. For 80 children, their parents provided at least one SES indicator, and for 45 children full information on all indicators was available. See Table 1 for an overview of the amount of children who had which combinations of SES indicators available for use. If the education and/or occupation of two parents were reported, the average was used. If information of only one parent was reported, this was used. Income was measured for the household as a whole (and only for one household), so that always consisted of a single score.

**Table 1:** Overview Provided Combinations of SES Indicators by Parents

Income	Education – mother	Education – father	Occupation – mother	Occupation – father	Number of children
Yes	Yes	Yes	Yes	Yes	45
No	Yes	Yes	Yes	Yes	6
Yes	Yes	Yes	No	Yes	8
Yes	Yes	Yes	Yes	No	7
Yes	Yes	Yes	No	No	9
No	Yes	Yes	No	Yes	1
Yes	Yes	No	Yes	No	1
Yes	Yes	No	No	No	1
No	No	No	No	Yes	1
Yes	No	No	No	No	1

Independent of parents’ answers, children were asked to estimate their family’s income with the question ‘How much money do you think your family has to buy expensive things?’ (answer scale (1) Very little – (5) A lot). In addition, for all parent(s)/caregiver(s), they were asked to indicate their education and occupation with the following questions: ‘How long do you think your mom/dad/caregiver studied after high school in the past?’ ((1) Not long at all – (5) Very long); and ‘What occupation do you think your mom/dad/caregiver has?’ (open-ended question). A follow-up question was: ‘To what group of occupations do you think this occupation of your mom/dad/caregiver fits best?, on a 5-point Likert scale using the same occupations for the answer options as mentioned in the former section.

#### SES group evaluations

Socioeconomic group evaluations with respect to income, education, and occupation were measured via fictitious new classmates, all of which had the same gender as the participants. Questioning preadolescents about their desire to befriend fictitious peers is an appropriate tool for examining their evaluations of (dissimilar) others (e.g., Thijs & Verkuyten, 2023). Children were asked to imagine six times that they had a new classmate, while seeing the two parents of this classmate via an illustration (see Appendix A). The two parents were described as: (1) “Parents who can buy many expensive things”, (2) “Parents who can almost never buy expensive things”, (3) “Parents who studied for a long time after high school in their past”, (4) “Parents who did not study after high school in their past”, (5) “Parents who have an occupation like doctor, dentist, or judge”, to signal high and low income, high and low education, and low and high occupational status, or (6) “Parents who have an occupation like gardener, cleaner, or window cleaner”, to signal high and low income, high and low education, and high and low occupational status respectively. The order of the six parental signals was randomized. For each new classmate, children were asked to answer the following statements; (1) ‘Would you like to become friends with this child?’, (2) ‘Would you like to have a play date at your house with this child?’, and (3) ‘Would you like to have a play date at the house of this child?’. All statements were answered on a 5-point Likert scale ranging from (1) No, definitely not, to (5) Yes, definitely. Principal component analysis indicated that these items loaded on one factor for each peer (with Eigenvalues > 1, and 71.56–84.90% explained variance). Hence, the scores for the three statements were combined into one scale; *group evaluation*, which has a high internal consistency^3^ (Cronbach’s alpha ranging from 0.879–0.927).

Finally, children were asked to select two of the new classmates – one they would most like to become friends with, and one they would least like to become friends with – and elaborate on this selection in an open question. Children’s given reasons were coded using qualitative content analysis. The analysis process consisted of categorizing parts of the material, and discovering themes from these categories (Cho & Lee, 2014). More specifically, the analysis can be considered a deductive directed content analysis; first, initial codes were constructed using relevant previous research findings as guidance (Hsieh & Shannon, 2005). The initial codes were: (1) Child’s or parents’ manners, (2) Preferences for activities, (3) Child’s or parents’ appearance, (4) Parenting style, and (5) Child’s or parents’ characteristics. Then, codes were refined and added (i.e., emergent codes from the data) during the analysis of the first 25 answers. The emergent codes were: (1) Preference for normal/similarity, (2) Empathy, and (3) Self-interest. See Appendix B for a description of all codes.

Intercoder reliability was obtained by having two independent raters coding 20% of the answers. After establishing a sufficient intercoder reliability of Cohen’s Kappa = 0.79 (i.e., a substantial agreement, Landis & Koch, 1977), the remaining answers were coded by only one of these raters (the first author). Some children chose more than one fictitious peer (although it was explicitly stated to choose only one). If children referred to these other peers in their answers, the codes for these parts of the answer were assigned to the other peers. If this was unclear, it was assumed that their elaboration applied to all chosen peers, giving them the same code.

### Planned Analyses

To test hypothesis 1, correlations between self-reported SES provided by parents and children were inspected for the three SES indicators. Hypothesis 1 was evaluated by inspection of the significance (*p* < 0.05) and magnitude of the correlations. An a priori power analysis was conducted using G*Power version 3.1.9.4 (Faul et al., 2007). The required sample size (power = 0.80, correlation H1 = 0.3, significance criterion = 0.05) was *N* = 67. As it was not possible to know beforehand how many parents would be willing to provide their SES, more students were recruited to be sure. For all SES indicators there is sufficient data, except for the occupational status of both parents – which is slightly less than 67.

To test hypothesis 2, six repeated measures ANOVAs were performed as *main analyses* to test differences between children’s estimations on a particular SES indicator for sets of parents that differed on another indicator, for example scores on education for high- versus low-income parents. The required sample size (power = 0.80, effect size f = 0.25, significance criterion = 0.008, correlation among repeated measures = 0.2) was *N* = 82. Hypothesis 2 was evaluated by inspection of significance (p < 0.008; see further details below) and effect sizes (η^2^). Further, as *exploratory analyses*, again the six repeated measures ANOVAs were performed. In a first step, children’s age was included as covariate to test whether age affected their understanding. In a second step, children’s parent-reported family SES indicators were included as three covariates to test whether this affected their understanding. Here, age was removed again as there is no interest in interaction effects between SES and age. For the exploratory part of this study, no power analysis was performed.

To test hypothesis 3, three repeated measures ANOVAs were performed as *main analyses* to test differences between children’s group evaluations for the low- and high-SES peer of a specific indicator. The required sample size (power = 0.80, effect size f = 0.25, significance criterion = 0.017, correlation among repeated measures = 0.4) was *N* = 53. Hypothesis 3 was evaluated by inspection of significance (p < 0.017; see further details below) and effect sizes (η^2^). Further, as *exploratory analyses*, again three repeated measures ANOVAs were performed with the three family SES indicators as estimated by the students *themselves* included as three covariates to test whether children’s group evaluations depended on this. Finally, as mentioned above, a deductive directed content analysis was performed to investigate why children showed SES biases.

Missing data in the ANOVAs was handled with listwise deletion to be able to compare results between the analyses with the same subsample. Hence, if children had no parent-reported SES data on one or more indicators, they were excluded – which led to a subsample of *N* = 61 for the exploratory analyses related to Hypothesis 2. For these exploratory analyses a robustness check was conducted, in which the SES indicators were separately added to the ANOVAs to be able to test their effects with a larger sample size (*N* = 69, *N* = 78, and *N* = 72 for occupation, education and income, respectively), which led to the same conclusions. With respect to the correlation analyses, missing data was handled with pairwise deletion.

In this study, many ANOVAs were conducted, among which four ‘families of tests’ are considered (i.e., main analyses H2, exploratory analyses H2, main analyses H3, and exploratory analyses H3). In the *main analyses* a Bonferroni correction was used to account for multiple testing, which means Hypothesis 2 and 3 are inspected for significance with a stricter *p*-value (for H2 the *p-*value of 0.05 is divided by six, for H3 this is divided by three; see above). As the other analyses are *exploratory* and already have a lower power due to a higher complexity of analyses, no correction was used there. This means the exploratory results should be interpreted with caution.

## Results

### To What Extent can Children Accurately Estimate Their Own Parental SES?

Correlations (see Table 2) were inspected to test whether children can estimate their own family’s SES and especially estimate their parents’ income and occupation (Hypothesis 1). For all SES indicators (i.e., income, mothers’ education, fathers’ education, mothers’ occupational status, and fathers’ occupational status) there were significant correlations between the SES indicator provided by parents or caretakers and as estimated by their child. The correlations for income and mothers’ education were considered weak to moderate, the correlation for fathers’ education moderate, and the correlations of both parents’ occupational status were considered moderate to strong (Cohen, 1988). This is partially in line with Hypothesis 1: Children indeed seemed especially able to estimate their parents’ occupation as these correlations were the strongest.

**Table 2:** Correlations Between SES Indicators, Provided by Parents (P) and Children (C)

	*N*	Range	M	*SD*	1.	2.	3.	4.	5.	6.	7.	8.	9.
1. Income (P)	76	1–5	3.37	0.73									
2. Income (C)	89	2–5	3.52	0.62	0.243*								
3. Education Father (P)	80	1–9	5.73	2.07	0.465**	0.065							
4. Education Father (C)	85	1–5	3.31	0.83	0.106	0.113	0.330**						
5. Education Mother (P)	82	1–9	5.84	1.86	0.317**	0.188	0.593**	0.169					
6. Education Mother (C)	86	1–5	3.43	0.83	0.011	0.050	0.294*	0.582**	0.244*				
7. Occupational status Father^a^ (P)	65	2–4	2.98	0.89	0.402**	0.043	0.544**	0.175	0.437**	−0.003			
8. Occupational status Father (C)	85	1–5	2.73	1.17	0.231	−0.001	0.427**	0.356**	0.403**	0.143	0.449**		
9. Occupational status Mother (P)	61	1–4	2.70	0.82	0.490**	0.155	0.500**	0.202	0.546**	0.183	0.537**	0.264	
10. Occupational status Mother (C)	86	1–5	3.23	1.39	0.127	−0.016	0.325**	0.074	0.447**	0.157	0.447**	0.308**	0.537**

^a^ = occupation provided by parents, and then coded into a scale from 1–5 using ISEI-08 scores. For education, parents gave their educational level on a scale from 1 (primary education) to 9 (doctorate). Children indicated how long their parents went to school after high school on a scale from 1 to 5. For income, both parents and children indicated this on a 5-point scale

** = correlation is significant at the 0.01 level

* = correlation is significant at the 0.05 level

Bold values identify statistical correlations

### Do Children Understand the Relations Between the Three Indicators of SES?

Six repeated measures ANOVAs were performed to test whether children understood that the SES indicators are related – especially income and occupation (Hypothesis 2). More specifically, it was tested whether children gave different estimates for a particular SES indicator for sets of parents that differed on another indicator, for example scores on education for high- versus low-income parents. In line with Hypothesis 2, all ANOVAs were significant with large effect sizes (Cohen, 1988; see Table 3), which shows that children generally think that people with a high SES on one indicator, also score higher on the other indicators compared to people with a low SES on the manipulated indicator. Moreover, the differences were symmetrical. For example, children assigned high-educated parents a significantly higher occupational status than low-educated parents (see final row Table 3), and parents with a high occupational status a significantly higher education than parents with a low occupational status (fourth row Table 3). When comparing effect sizes, the relation between income and occupation (as expected) but also the relation between education and occupation seems most clear for children. The relation between income and education seems to be a bit less clear for children, as one fourth of the children had zero or negative difference scores for high versus low SES. Yet, even for this relation the effect sizes were large, hence overall children see strong relations between the socioeconomic indicators income, education, and occupation.

**Table 3:** Repeated measures ANOVA for difference between high- and low-SES on other SES indicators

	Mhigh-SES	*SD*high-SES	Mlow-SES	SD low-SES	Mean diff.	% -diff.^a^	% 0 diff.^b^	% + diff.^c^	F	η^2^
Rich vs Poor - occupation	3.98	0.95	1.40	0.79	2.58	3.4	1.1	95.5	328.64***	0.79
Rich versus Poor - education	3.74	1.07	2.25	0.96	1.49	13.5	10.1	76.4	72.2***	0.45
High vs Low occupation - income	4.06	0.63	2.65	0.68	1.41	0.0	12.4	87.6	216.26***	0.71
High vs Low occupation - education	4.46	0.76	2.54	0.84	1.92	2.2	5.6	92.1	208.83***	0.70
High vs Low education - income	3.63	0.68	2.34	0.75	1.29	2.2	23.6	74.2	132.88***	0.60
High vs Low education - occupation	3.82	0.97	1.70	0.88	2.12	3.4	9.0	87.6	221.25***	0.72

High difference scores suggests a better understanding of the relation between the two indicators

^a^ = % children with negative difference score

^b^ = % children with no differences in scores

^c^ = % children with positive difference score

*** = *p* < 0.001

For the *exploratory analyses*, in a first step (*N* = 89), children’s age was added as covariate to the six ANOVAs to explore whether children’s understanding depended on their age. For all ANOVAs, no interaction effects between the difference in estimations on any SES indicator and children’s age was found (with *η*^*2*^ ranging from 0.002 - 0.04), which indicated that age did not affect their understanding about the relations between SES indicators.

In a second step (*N* = 61), children’s own family SES indicators (parent-reported income, education, and occupational status) were separately added as covariates to the six ANOVAs to explore whether children’s understanding about the relations between SES indicators depended on those factors. Potentially, 18 significant interaction effects could have been found, but there are only two significant interaction effects. That is, only the ANOVA in which the difference in income estimations between high- and low-status occupations was tested, showed two effects. The *higher* the parents’ educational level, the larger the perceived difference in income between parents with a low- and high-status occupation (*F*(1,1) = 6.25, *p* = 0.015, *η*^*2*^ = 0.10). At the same time, the *lower* the parents’ occupational status, the larger the perceived difference in income between parents with a low- and high-status occupation (*F*(1,1) = 4.98, *p* = 0.030, *η*^*2*^ = 0.08).

### How do Children Evaluate Peers with High- and Low-SES Backgrounds?

Table 4 provides descriptive statistics, with more in-depth descriptives showing that there were different patterns of biases for each SES indicator. First, for *income and occupation*, there was no clear socioeconomic bias toward either the high- or low-SES group as – besides children showing no bias at all – children were almost equally divided between having a bias for the high- or for the low-SES group. However, for *education*, there was a different pattern; again quite some children showed no bias, but if they showed a bias, it mainly manifested as a preference for peers with high-educated parents. Notably, on all SES indicators relatively many children showed no bias at all^4^. Table 5 contains the correlations between the different biases (obtained by subtracting the evaluations of the high- versus low-SES peers). The bias for occupation was not correlated with bias for education, nor for income (see Table 5). Only the biases for income and education were weakly to moderately correlated. This underscores that children differentiate between the SES indicators in their biases.

**Table 4:** Descriptive statistics for group evaluation measures

	*M*_low-SES_	*SD*_*low-SES*_	*M*_*high*-SES_	*SD*_*high-SES*_	No bias^a^	Preference for high-SES	Preference for low-SES
Income^b^	3.36	0.96	3.22	1.08	27%	33.7%	39.3%
Education^c^	3.19	0.81	3.58	0.82	42.7%	44.9%	12.4%
Occupation^d^	3.41	0.94	3.32	1.01	34.8%	27%	38.2%

^a^ = no bias indicates a difference of zero between peer with high- and low-SES

^b^ = difference score between preferences (average of the three questions) for rich and poor fictitious peer ^c^ = between peer with high- and low educated parents

^d^ = between peer with high and low occupational status parents

**Table 5:** Correlations between biases of different SES indicators

	*N*	Range	1.	2.
1. Income bias	89	−3.33 to 3.67		
2. Education bias	89	−1 to 3.0	0.257*	
3. Occupation bias	89	−3.0 to 3.0	−0.027	0.005

* = correlation is significant at the 0.05 level

Three repeated measures ANOVAs were performed to test whether children show SES bias in their group evaluations – in which it was expected that children show a positive bias toward peers with high-educated parents (Hypothesis 3). In the ANOVAs it was tested whether children showed significant differences in their group evaluations for the fictitious peer with a low- and high SES on the same indicator (i.e., rich versus poor, high- versus low-educated, and high- versus low- occupational status). Whereas the ANOVAs for income (F(1, 1) = 0.58, p = 0.447) and occupational status (F(1, 1) = 0.60, p = 0.441) showed no significant differences within children in their group evaluations, the ANOVA for education showed a significant difference (F(1, 1) = 23.33, p <0.001, *η*^*2*^ = 0.210). This means that children showed a positive bias for the peer with high-educated parents, compared to the peer with low-educated parents, which is in line with Hypothesis 3.

In a second step (*N* = 89), children’s own SES was added, and in line with the intergroup perspective the *child-reported* parental income, education, and occupational status were used instead of the parent-reported SES indicators. These measures were added as covariates to the three ANOVAs to see whether children’s own perceptions of their SES affected their socioeconomic biases. For all ANOVAs, no interaction effects between the difference score of the estimations and (one of) the covariates were found, which indicated that children’s own perceptions of their SES did not affect their group evaluations.

### What are Children’s Reasons for Their Preferences for Certain Fictitious Peers?

The open answers children gave to elaborate on their choice of the peer they wanted to befriend *most* were examined. Most children chose the peer with high-educated (*n* = 28), rich (*n* = 23), or poor (*n* = 21) parents as the peer they most wanted to become friends with (see Appendix C for the coding scheme). Children gave different reasons for selecting those peers. First, with respect to the peer with *high-educated parents*, most children referred to personal reasons, that is to say self-interest and a preference for ‘normal’. Thus, children indicated that they could benefit from the parents’ or peers’ knowledge (e.g., “I think it is nice, because then I can maybe do my homework with her together”, or “those parents can teach me something”) and that high-educated parents seemed most normal to them (e.g., “Because they have somewhat normal parents. The rest seem less so to me”). Sometimes children specifically referred to a “normal” income, even though the family income of the peer was not given (e.g., “He is a normal person. He is not someone who thinks he is more important and richer than another person who is not rich”).

Second, regarding the peer with *rich parents*, most children referred to self-interest (one of the personal reasons), which always involved material and wealth aspects. For example, children mentioned that “If they for example live very large, then you are often allowed to do a lot and then you can often go shopping and eating out with them”, or “Then they always have something special with them”.

Finally, for the peer with *poor parents*, most children chose this peer out of empathy. They gave answers like “Well, I think if you are poor you do not have many friends, so then I want to become friends with him because he does not have many”, or “Well, then you can help her family, and if she really wants something but cannot afford it and you can, then you can buy it for her”. Another reason to choose the poor peer was because of the child’s characteristics. For example, they wanted to befriend the poor peer because “That boy would be very grateful for everything he gets”, or they thought that he/she was “not spoiled and seems nice”.

Then, the open answers children gave to elaborate on their choice of the peer they wanted to befriend *least* were examined. Most children chose the peer with rich (*n* = 36) or poor (*n* = 21) parents as the peer they least wanted to become friends with (see Appendix C) – again for different reasons. With respect to the peer with *rich parents*, most children referred to the child’s manners (i.e., bragging and acting mean). They gave answers such as “Because the boy is then probably very spoiled, and because of that he can be very bossy or mean”, or “Because she can brag the whole time about all the new stuff she got from her parents”, or “Well probably she will be very mean because she has a lot of money and if I, for example, did not have a lot of money I think she is going to bully you because you do not have money”. Some children referred to the same manners, applied to the parents instead of the child (i.e., they are arrogant, they will brag about their money).

Regarding the peer with *poor parents*, most children referred to appearances, either of the parents or the child. They gave answers such as “Because she does not smell nice and look neat”, or “she has old clothes”, or “I don’t like to see it that those parents do not have a lot of money”. A few children gave reasons out of self-interest; “Because they don’t really have fun stuff to play with”.

## Discussion

Although there is ample research showing the effects of parental income, education, and occupation on children’s lives (e.g., Klein et al., 2020), which implies the need to consider all the three indicators in research, very little is known about children’s understanding of SES as a multidimensional construct including income, education, and occupation. Further, little is known about children’s group evaluations based on these three SES indicators. The current study aimed to address this research gap by taking into account parental income, education, and occupation to see whether children are aware of their family’s position on these indicators, understand that these indicators are related, and show similar group evaluations across them. It was found that children’s estimations of their own family SES corresponded only moderately to the SES reports of their parents. Moreover, children understood that income, education, and occupation are related, but at the same time they distinguished between these indicators in their (hypothetical) peer evaluations. That is, they showed a clear preference for peers with high-educated parents compared to peers with low-educated parents, whereas for income and occupational status there was no clear overall bias. Thus, differences between SES indicators are meaningful for children, which emphasizes the importance of considering all three SES indicators separately when studying children’s ideas about SES.

### Children’s Estimations of their Own Family SES Indicators

It was hypothesized (Hypothesis 1) that children would especially be able to accurately estimate their parents’ occupation and income, compared to their parents’ education. This is partially confirmed, as children are indeed more strongly aware of their parents’ occupational status – but education and income were more difficult for them to estimate. This might be because education and income are, compared to occupation, more abstract and children’s abstract thinking still develops in late childhood (Simons & Keil, 1995). Also, unlike their occupation, parents’ education is something from the past, which may make it less salient for children. Ackerman and Elenbaas, (2023) who found no correlations between children’s SSS ratings and parent-reported *income*, argued that resource and lifestyle cues are not always informative of parents’ income. For example, one study found that mothers with a low income reported buying their children nicer clothes and toys to convey a sense of stability (Mistry & Lowe, 2006). This might explain the weak correlation between parent-reported income and children’s estimated income in the present study. Presumably, following CDP, when children grow older and turn into adolescents, they become better able to estimate their own family SES – it has already been shown that perceptions of SSS of late adolescents (Goodman et al., 2015) are more aligned with parent-reported SES than preadolescent children’s perceptions (Mistry et al., 2015).

### Children see Relations between SES Indicators

Children in the current study understood that people with a high SES on one indicator, also have a higher SES on the other indicators. The SES indicators thus seem related to one another not only in real life (Bollen et al., 2001), but also in children’s minds. This is in line with the *cognitive developmental perspective* (CDP; Furth, 1980) and *social representations theory* (SRT; Moscovici & Hewstone, 1983). Whereas the CDP would claim that preadolescent children have the cognitive capacities to understand the ‘logical’ relationships between different SES indicators, SRT would state that they are exposed to social representations of these relationships in their daily lives. Children saw especially strong relations between occupation and income, in line with Hypothesis 2 and previous research (e.g., Vandebroeck, 2021), and between occupation and education. The perceived relation between education and income was strong as well but comparatively less evident– and for around one fourth of the children it was not positive. Following CDP, this might be because the relation between education and income is more indirect, thus less “logical” and therefore harder to understand. Following SRT, this might be because children are probably less likely to “come across” this relation than the relations between education and occupation, and between occupation and income, because there are stronger social representations of the latter. For example, a high-educated person in a movie is typically a professor or inventor but not necessarily rich. Another reason in line with SRT, might be that they struggle more with this relationship because of the societal context of the Netherlands, with a relatively weaker endorsement of meritocratic beliefs (Mijs, 2018) which makes it less evident that a higher education leads to more money.

In the current study, children’s own SES and age were generally not related to their understanding of the relation between socioeconomic indicators. This suggests that most preadolescent children understand the relations between income, education, and occupation in the same way – regardless of their own age and SES background. Presumably, following SRT, there is a shared social context (for example through school or social media) with social representations in it through which *all* children gain this understanding. However, it is important to note here that the current study had a small age range, thus it might well be that when children grow older and turn into adolescents, they see stronger connections between socioeconomic indicators than found in this study (see Vandebroeck, 2021). There was one exception in which children’s own SES was related to their understanding. Children of parents with a *higher* educational level, but also of parents with a *lower* occupational status, reported larger income gaps between families with low- and high status occupations (and thus a stronger association between occupation and income). A previous study also showed that children’s own SES influenced their understanding, yet they found contrasting results – namely that students with parents with a *higher* occupational status expected larger earning differences between high-status and low-status occupations (Dräger & Wicht, 2021). Yet, participants in that study were older (ninth-graders and further), which might explain the discrepancy. Overall, this suggests that children’s own SES might influence their socioeconomic understanding to some extent, but it is unclear yet how it does – as findings thus far seem contradictory, and findings from this part of the current study should be interpreted with caution.

### Children Show Differentiated Socioeconomic Group Evaluations

Children’s socioeconomic group evaluations were not influenced by their own family SES – according to their own estimations. Thus, for example, for their group evaluations for parents with a low- and high occupational status it did not matter what children thought the occupational status of their own parents was, although children’s estimations of their family SES indicators were correlated with their actual family SES. Moreover, even though the SES indicators were strongly related to one another in the minds of the children in this study, they showed differentiation in their group evaluations for the different SES indicators. It was expected that children would show a positive bias for peers with high-educated parents, compared to peers with low-educated parents (Hypothesis 3), which was confirmed. In contrast, for income and occupation, children did not show a clear overall bias toward either low- or high-SES groups. Also within children there was differentiation in biases for the SES indicators, as those biases were not or not strongly correlated to each other. This shows that children differentiate between the SES indicators in their group evaluations. This underlines the fact that it is necessary to take SES indicators separately into account (and especially education and income) when studying students’ ideas about SES. Also, this supports the need for more research among children taking multiple dimensions of SES into account by looking at the different indicators, which rarely has been done to date.

### Education bias

As expected, children showed a clear positive bias toward peers with high-educated parents, which might be due to the fact that high-educated people tend to have a higher social standing in the Netherlands (Kuppens et al., 2018), with currently a lot of attention for this. It is therefore important for future research to investigate whether these findings are generalizable to other societal contexts. The peer with high-educated parents was also relatively often chosen (by 28 of the 89 participants) when children were asked to select the peer they would like to befriend the most. When they elaborated on their choice, they often mentioned that this peer seems most normal to them compared to the other peers. Also, children often gave reasons for this related to self-interest, they for example wanted to benefit from the parents’ or peers’ knowledge (e.g., getting help with homework). Possibly, children were aware that if they obtained good grades at school, they had a higher chance of educational success. This might especially be the case for children in grades 4–6, which in the Dutch school system implies a period of important educational decision making, and the grades children obtained at school highly influence this decision. This might suggest that children show a desire to belong to the high-status group, by benefitting from friendships with peers with high-educated parents.

### Occupation and Income bias

Both for occupation and income, children did not show a clear overall bias toward either the high or low-SES peer. However, with respect to income bias, the rich and poor peer where often chosen in children’s choices for the peer they would like to befriend the most or the least (i.e., number of times chosen (out of 89): most friends, poor = 21, rich = 23 and least friends, poor = 21, rich = 36). This suggest that for many children income is, compared to occupational status and education, the most important socioeconomic indicator in their friendship preferences. Moreover, unlike occupational status and education, children showed clear stereotypical ideas about people based on their income, which differed for peers with rich versus poor parents. When children explained why they did not want to befriend a *rich* peer they referred to the child’s or parents’ characteristics, stating that rich people are mean, arrogant, and always bragging about their money and possessions (in line with previous research, e.g., Elenbaas et al., 2022). In contrast, when children did not want to befriend a *poor* peer, they described the peer and/or their parents as not neat, and not smelling and looking nice. If children preferred to befriend a *rich* peer this was almost always out of material self-interest. In contrast, if children preferred to befriend a *poor* peer they displayed empathy, as they felt sorry for them and reported a desire to help them.

Our findings align with a Belgian study (Kostet et al., 2022) on children’s ideas about economic inequality, where children tended to express empathy toward poor people, and attributed negative features (i.e., greedy, selfish) to rich people. The researchers argued this might partially be explained by the fact that Belgian society is based on principles of equity instead of a liberal welfare regime with meritocratic ideas which is more prominent in the U.K. and the U.S. Interestingly, in studies conducted in societies in which people hold more meritocratic beliefs, children tend to have more positive stereotypes toward rich people (e.g., Mistry et al., 2015). Hence, the results regarding children’s stereotypes toward rich and poor people in the present study might be explained by the Dutch societal context. This is in line with research showing that people’s prejudice toward poor and rich people depend on their beliefs about the causes of and control over poverty – whether those are external or internal (Fiske et al., 2002). Research into children’s explanations and ideas of economic inequality shows that U.S. children view being rich and poor as caused by factors within a person’s control (e.g., because they do not work or because they do not try hard enough; Mistry & Yassine, 2022). Thus, they tend to emphasize individualistic rather than structural explanations for inequality (Sigelman, 2012). To further unpack this, future research can test whether children’s meritocratic ideas, which are associated with negative stereotypes toward the poor among adults (Godfrey & Wolf, 2004), influence stereotypical ideas of rich and poor peers.

### No Bias

At the same time, it is worth noting that for each SES indicator a considerable group of children (around one third) showed no bias at all. Children’s open-ended answers suggested that one reason for this was that they did not want to judge their peers based on parents’ characteristics (i.e., “I don’t care, it depends on whether the girl is nice”). Children differentiated in their biases between the SES indicators, which means that if children showed no bias for one of the SES indicators, this did not always imply that they also showed no bias for the other SES indicators. Therefore it is unlikely that children showed no bias out of social desirability, as one would then expect that they show no bias on all three indicators.

### Intergroup Perspective

The socioeconomic biases in the current study do not reflect an ingroup preference, as children showed no positive bias toward peers with parents with a similar SES, which is in line with two other studies taking children’s SSS into account (Ackerman & Elenbaas, 2023; Mistry et al., 2015). Although Developmental Intergroup Theory (Bigler & Liben, 2007) and Social Identity Development Theory (Nesdale 2004) argue that children this age have the capacity and are inclined to categorize themselves and others as group members, and are motivated to prefer their ingroup over their outgroup(s), the present study shows that at least the latter does not hold for SES. Thus, even when in the current study artificial contrasting SES categories were created through the vignettes, still children did not use SES as categorization with clearly defined groups in which they can place themselves and others (in other words, they made no clear ‘us versus them’ distinction based on the SES indicators). While at the same time, children in the current study had associations and stereotypical ideas about contrasts on the SES indicators (e.g., rich versus poor).

SES indicators are, as opposed to gender and ethnicity, continuous rather than nominal, which might explain why children do not consider clear in- and outgroups with respect to SES. Possibly, however, when children turn into adolescents, their own SES will be increasingly important for them, as it will become more and more their *own* SES instead of their *family* SES. In the Netherlands, this starts when children enter secondary education as they become part of a group with the same educational level then. This might mean, for example, that adolescents at higher educational levels will become negatively biased toward those at lower educational levels (cf., Kuppens et al., 2018). In other words, it might be that in contrast to the preadolescents in our study, adolescents develop ingroup preferences in their group evaluations with respect to the SES indicators income, education, and occupation.

### Limitations and Directions for Future Research

There are three limitations worth noting. First, the sample was relatively small and, although heterogeneous in terms of family SES, it consisted almost exclusively of ethnic majority children. Further, 23.6% of the children were excluded from the study as they had no parental consent, which might be because the SES questionnaire discouraged parents from giving consent. Consequently, children without consent might mainly be children with a rather low or high SES, leading to a bias in the sample which might have affected the results. This could have affected the correlations between the child- and parent estimations of SES due to a restriction of range (Wiseman, 1967), although it should be noted that there was still sufficient variation in the measures (see Table 2). Moreover, based on contact theory (Allport et al., 1954), it might be argued that children in more socioeconomically homogeneous classes show more socioeconomic biases. Accordingly, it is important to further investigate whether the present findings hold for a larger, more representative sample consisting of children from different types of schools.

Second, children in the current study were prompted to think about specific socioeconomic groups on two extreme positions (e.g., poor versus rich), and it remains to be seen whether they spontaneously evaluate groups based on SES in their daily lives – as this cannot be inferred from this study. Naturally, in reality children evaluate peers based on many different dimensions simultaneously which is very different than the hypothetical scenarios in this study in which children evaluated a peer based on only one dimension. Moreover, all racial and family-type stimuli were kept constant, which also does not align with reality. Yet, this made it possible to examine the isolated, separate effects of the SES indicators on children’s group evaluations.

Finally, the way in which children were asked to estimate their parents’ educational level (i.e., ‘How long do you think your mom/dad/caregiver studied after high school in the past?’) did not completely align with the way in which we asked parents about their own educational level (i.e., their highest obtained educational level). Although we made this choice for the sake of comprehensibility for children, it might have affected the correlations between children- and parent-reported educational level. Further, we asked parents about their income via a rough estimation, which might have picked up less on actual income differences. This measure was also quite subjective, as wealthier people might have higher expectations of what constitutes ‘making ends meet’ and consider fewer things to be expensive. Although we made this choice to avoid that parents felt a resistance to fill in the questionnaire because of too intimate questions, it might have affected the results regarding its effect on children’s socioeconomic understanding, as well as the correlations between children- and parent-reported income.

### Implications for Research and Practice

It is important to consider the results of the current study when researching effects of SES on (pre-) adolescents, as it emphasizes that the SES indicators income, education, and occupation cannot be interchangeably used. Research to date often combines them into one variable, or considers only one of the indicators, whereas this study shows that they elicit different group evaluations among children and are not always strongly correlated. Researchers need to carefully consider which mechanisms are at play in their research, and which indicator(s) are then important to include – as it cannot be assumed that they will have similar effects. Especially, it is important to differentiate between income and education as children seem to use them differently to form their preferences, and the relation between these two indicators is least clear for them compared to the relations between the other indicators. Further, our findings show that it is important to take occupational status and education – besides only income – into account when studying children’s socioeconomic biases and stereotypes.

From a practical perspective, our findings show that preadolescent children have biases and stereotypes based on family income and educational level, and those could be targeted in interventions. To date, there has been little attention for children’s socioeconomic biases in educational policies and interventions, although there is a considerable literature on ethnic diversity education (i.e., multicultural education; Banks & Banks, 2019). The current study suggests that socioeconomic differences could be considered as well.

## Conclusion

To date, very little was known about children’s understanding of SES as a multidimensional construct including income, education, and occupation, and about children’s group evaluations based on these three SES indicators. The present study examined children’s socioeconomic understandings *as well as* group evaluations based on income, education and occupation in another context than the U.S. or U.K. Its findings show that children see relations between the SES indicators, and at the same time show differentiated group evaluations between them, which shows that differences between SES indicators are meaningful for children. They show a clear positive bias for peers with high-educated parents compared to peers with low-educated parents, whereas for income their bias is not clearly toward either high- or low-SES; there seems to be one group of children with a positive bias for the poor peer, and one group for the rich peer. These results emphasize the importance of considering all three SES indicators separately when studying children’s ideas about SES.

## Figures and Tables

**Fig. 1:**
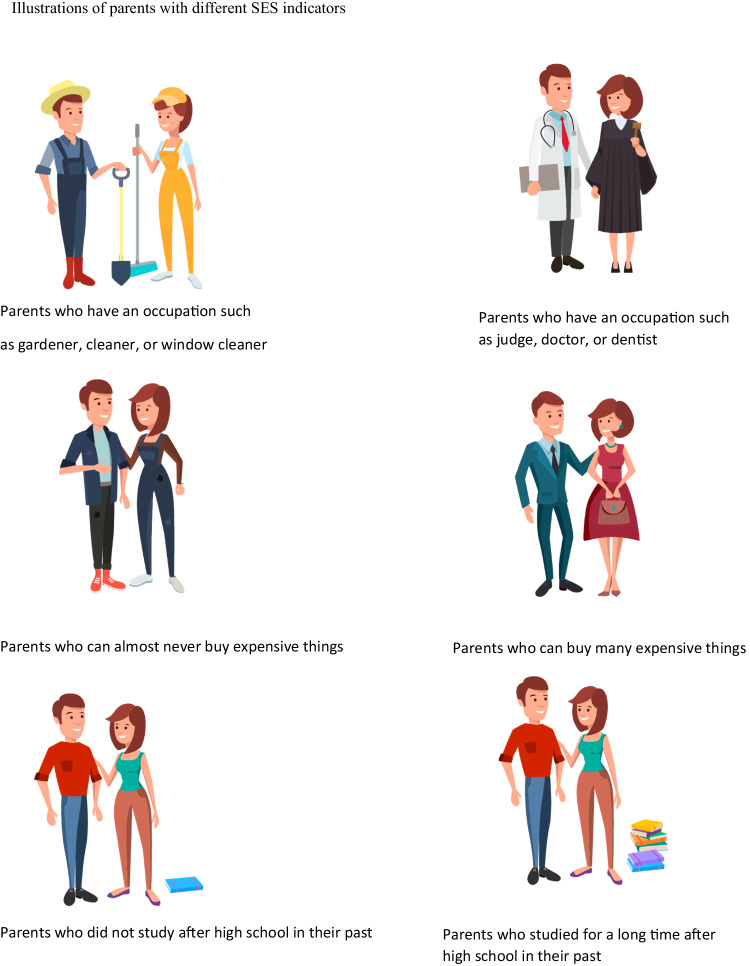
Illustrations of parents with different SES indicators

## Appendix A

Figure 1

## Appendix B

Table 6

**Table 6:** Initial and Emerged Coding Categories

Description		Example from data
**Initial codes – related to SES**^**a**^		
Someone’s manners	References to how someone *behaves*	About peer: “Well, if she comes to play she will act spoiled”
Preferences for activities	References to hobbies, sports, clubs etc.	About parents: “I love animals, and a gardener has something to do with animals”
Someone’s appearance	References to clothing, hair, language style etc.	About peer: “She has older clothes”
Parenting style	References to rules, atmosphere, norms etc. at the child’s house	“Because often very rich parents are pretty strict”
Someone’s characteristics	References to how someone *is*	About peer: “Usually very rich children are arrogant, and the poor are nicer”
**Emergent codes**		
Preference for normal / similarity	References to preference for normal or similar peers	“Because then they have somewhat normal parents. The others seem less nice to me”
Empathy	References to emphatic concern, feeling sorry	“Otherwise it is sad for the child that he has no friends”
Self-interest	References to personal interest or gains	“Maybe he will buy something for me”
**Other**		
Does not matter	Indicate to have no preference, do not care	“I actually do not care, as long as the girl herself is nice”
I don’t know	Indicate to not know why they have their preference	“I actually do not know”
No elaboration	No explanation or reason for their preference	“He just seems like a good boy”
Unclear	Not clear what is meant	“Just expensive”

*Note*. ^a^ = for these codes, we distinguished between references to parents’ and peers’ indirect indicators of SES – except for parenting style

## Appendix C: *Codes Reasons Friendship Preferences Fictitious Peers*

Tables 7, 8

**Table 7:** Elaborations Why Children *Most* Want to Become Friends with Chosen Fictitious peer

	Child					Parents						Emerged			Other
Chosen peer (*n*)	Character istics	Manners	Activities	Appearance	Total child	Character istics	Manners	Activities	Appearance	Parenting	Total parents	Empathy	Self-interest	Normal / Similarity	
Poor (21)	**7**	1			8	1			1		2	**10**	1	1	2
Rich (23)	2	1	2	1	6	1		1	1	1	4		**8**		9
Low-educated (8)	1				1						-			1	7
High-educated (28)	4				4	4	3				7		**6**	**6**	11
Low status occupation (12)	2				2	2		2	2		6			**3**	3
High status occupation (9)					–	1	2				3		**4**		5

**Table 8:** Elaborations Why Children *Least* Want to Become Friends with Chosen Fictitious peer

	Child					Parents						Emerged			Other
Peer (*n*)	Character istics	Manners	Activities	Appearance	Total	Character istics	Manners	Activities	Appearance	Parenting	Total	Empathy	Self-interest	Normal / Similarity	
Poor (21)	2			3	5		1		**4**		5		**5**		7
Rich (36)	2	**13**		2	17	5	4		1	1	11		1		9
Low-educated (13)	1	2			3	3	2	1			6		2		4
High-educated (6)	2	1			3	2					2				3
Low status occupation (8)		1			1			1			1				6
High status occupation (11)		2			2	1			1	**3**	5				5

## Footnotes

1 In the Netherlands, primary education ends at Grade 6, when children are generally 12 years old. Secondary education is divided into three broad tracks based on children’s academic achievement, which provide differential access to higher education or directly lead to the labor market (see for more information Luijkx & De Heus., 2008).

2 The mean differences between the estimations of the different occupation categories were all significant in the expected direction, except between the estimation of money bags for an occupation like ‘police offer, store owner, or dental assistant’ and ‘writer, pilot, or teacher’.

3 There were some small differences in children’s answers regarding where they preferred to have the playdate with the fictitious peer (their own or peers’ house). Specific findings are available upon request from the first author.

4 The cut-off value here is quite strict, hence a robustness check was conducted in which a value of -0.33 or 0.33 is still considered as *no bias* (as this means that on only one of the three questions a different answer was given that was 1 point lower or higher. This resulted in the same patterns).
